# Pulsed field ablation as a feasible option for the treatment of epicardial left ventricular summit premature complex foci near the coronary arteries: a case report

**DOI:** 10.1093/ehjcr/ytae478

**Published:** 2024-09-10

**Authors:** Dylan Spenkelink, Harry van Wessel, Vincent J van Driel, Hemanth Ramanna, Jeroen F van der Heijden

**Affiliations:** Department of Cardiology, Haga Teaching Hospital, Els Borst-Eilersplein 275, 2545 CH The Hague, The Netherlands; Department of Cardiology, Haga Teaching Hospital, Els Borst-Eilersplein 275, 2545 CH The Hague, The Netherlands; EF Department, Abbott Medical Nederland B.V., Veenendaal, The Netherlands; Faculty of Technology, Innovation and Society, University of Applied Sciences of the Hague, The Hague, The Netherlands; Department of Cardiology, Haga Teaching Hospital, Els Borst-Eilersplein 275, 2545 CH The Hague, The Netherlands; Department of Cardiology, Haga Teaching Hospital, Els Borst-Eilersplein 275, 2545 CH The Hague, The Netherlands; Faculty of Technology, Innovation and Society, University of Applied Sciences of the Hague, The Hague, The Netherlands; Department of Cardiology, Haga Teaching Hospital, Els Borst-Eilersplein 275, 2545 CH The Hague, The Netherlands

**Keywords:** Case report, Left ventricular summit, Premature ventricular complex, Pulsed field ablation, Irreversible electroporation

## Abstract

**Background:**

Radiofrequency catheter ablation in the left ventricular summit region is a challenging procedure due to proximity to the coronary arteries. Pulsed field ablation, a novel non-thermal ablation modality, does not cause damage to coronary arteries and may be used in the left ventricular summit region.

**Case summary:**

We describe a 45-year-old symptomatic patient with epicardial left ventricular summit premature ventricular complexes. Successful ablation of the focus was achieved by pulsed field ablation via a subxiphoid epicardial approach. Radiofrequency ablation would most likely have been ineffective due to the epicardial fat layer and potentially unsafe due to the proximity to the coronary arteries. Six months after ablation, the patient was asymptomatic and without ventricular ectopy.

**Discussion:**

For the first time, epicardial pulsed field ablation was successfully used for ablation of left ventricular summit extrasystole, where radiofrequency ablation could not be used because of the proximity of the coronary arteries. We conclude that pulsed field ablation might be a feasible option for this indication.

**Learning points**

- Left ventricular summit premature complex foci are challenging to treat with epicardial radiofrequency ablation due to proximity to coronary arteries and lesion formation hampered by an epicardial fat layer.
- Pulsed field ablation is a feasible option for catheter ablation of premature ventricular complexes originating from an epicardial focus, in particular the left ventricular summit.

## Introduction

Premature ventricular complex (PVC) is a commonly observed cardiac arrhythmia.^1^ Catheter ablation is a recommended first-line therapy for PVCs originating in the right ventricular outflow tract (OT) (Class I); however, the recommendation is lower (Class IIa) for radiofrequency ablation (RFA) of PVCs in the left ventricular (LV) summit due to increased risk of procedural complications.^2^ The LV summit is a triangular region situated in the most superior section of the left epicardial ventricular region, flanked by the two branches of the left coronary artery (CA) with the left main bifurcation as its apex, and covered more or less by epicardial isolating layers of fat.^3^ Mid-myocardially and epicardially located PVCs in the LV summit can be ablated from the cardiac venous system, the right or left OT, aortic cusps, and as a last option via an epicardial subxiphoid puncture.^3,4^ However, application of RFA via the subxiphoid route is often not possible due to the risk of damaging the CAs, or RFA is unsuccessful due to the inability to sufficiently reach the focus of the ventricular ectopy through the epicardial fat layer.^4–6^ The recommendation is to avoid RFA within 5 mm of the CAs.^7^

Pulsed field ablation is a novel ablation modality that induces cell apoptosis through applying a train of irreversible electroporation (IRE) pulses.^8^ Unlike RFA, PFA is a non-thermal ablation modality highly specific for cardiac muscle, resulting in less collateral damage in other tissues (for example CA and phrenic nerves, among others). Therefore, in contrast to RFA, an IRE pulse can be applied near CAs without potentially damaging them.^9,10^

In this case report, we describe a successful epicardial PFA of a LV summit PVC focus near the CA, where RFA was deemed unsuitable.

## Summary figure

**Figure:**
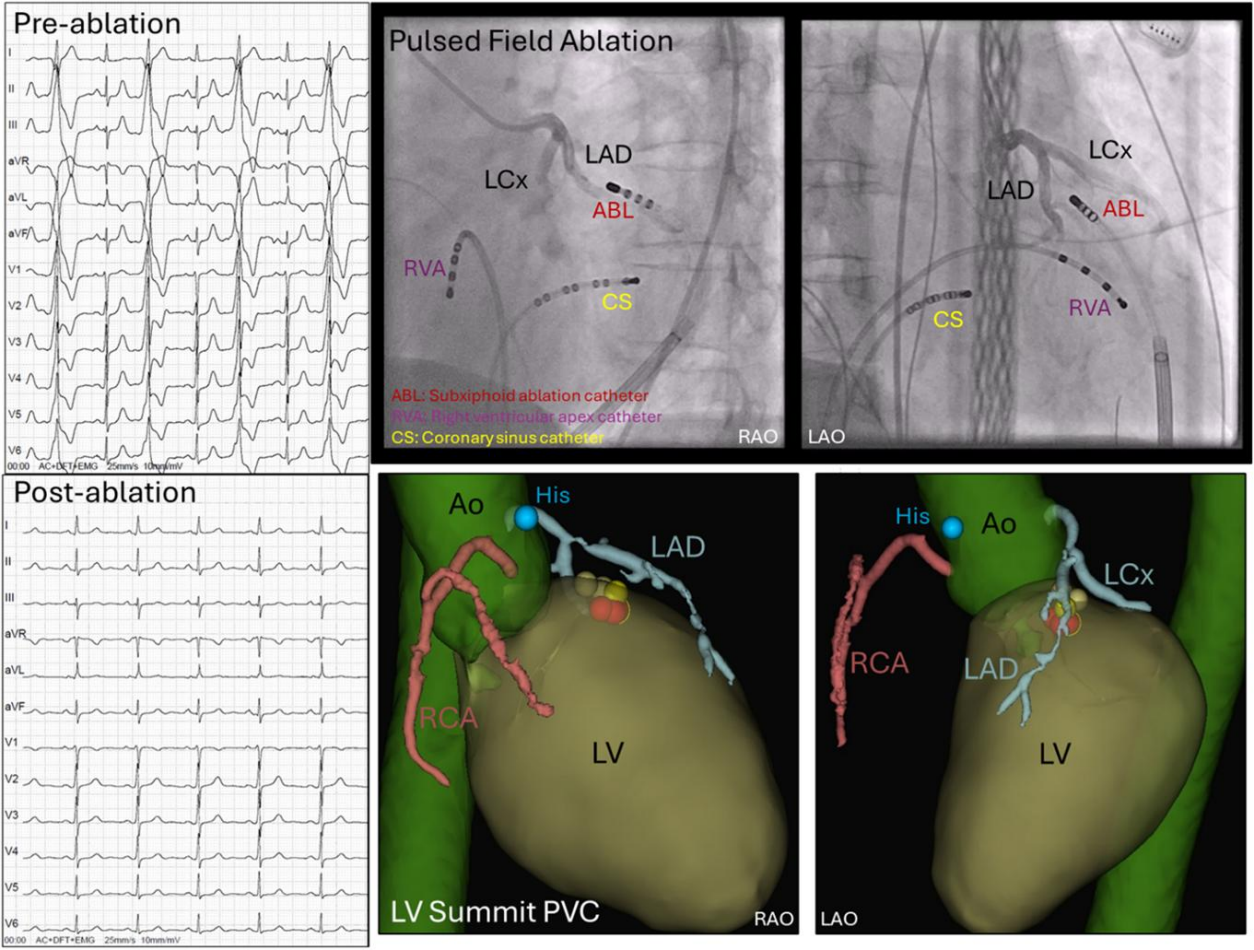
Case: a 45-year-old female. Successful pulsed field ablation (PFA) of epicardial LV summit PVCs via a subxiphoid epicardial approach. Pre- and post-ablation electrocardiograms of the patient in PVC bigeminy and sinus rhythm, respectively. The coronary angiograph shows the proximity of the ablation catheter positioned at the PVC focus to the CAs. This is confirmed by the 3D post-procedure segmentation indicating that the ablation lesions were applied near the CAs.

## Case presentation

**Figure 1:**
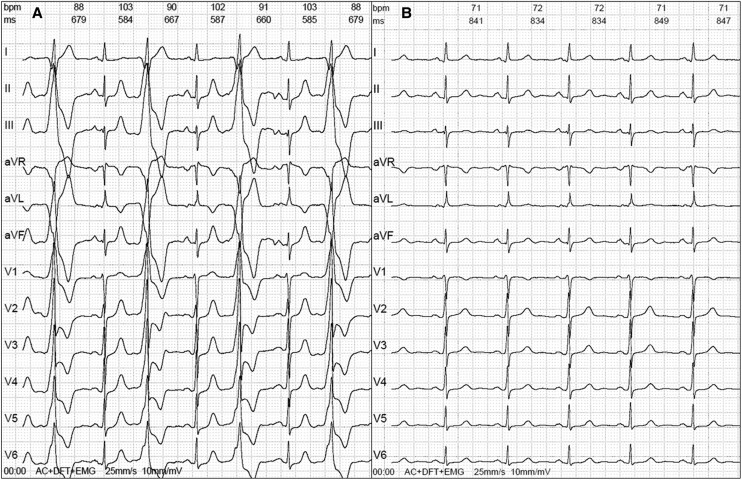
(*A*) Twelve-lead electrocardiogram of the patient before ablation with premature ventricular complex bigeminy. (*B*) Twelve-lead electrocardiogram of the patient 6 months after the third and successful ablation attempt in sinus rhythm.

The patient was a 45-year-old female with highly symptomatic PVCs. She had no past medical history or medication use of relevance. The baseline electrocardiogram (*Figure 1A*) suggested that the PVC focus was in the LVOT and a Holter indicated 50% PVC burden. Echocardiogram was normal. Suppression of the PVC with either flecainide or esmolol was unsuccessful; therefore, an ablation was recommended.

She underwent two unsuccessful ablation attempts before the successful procedure. The first procedure was performed without general anaesthesia with an endocardial approach. No early electrical signals were found endocardially in the anterior LV or LVOT. However, an early electrical signal was found in the great cardiac vein, cannulated with a 2-French mapping catheter (EPstar Fix 2F, Japan Lifeline, Japan). Advancing the ablation catheter (TactiCath™, Abbott Laboratories, IL, USA) to the point of earliest activity was not possible, and the distance between the tip of the catheter and the PVC focus was deemed too far for effective RFA. In the second procedure, a subxiphoid approach was planned, but after induction of general anaesthesia with initially sevoflurane followed by propofol, the PVC was absent. Attempts to induce the PVC with isoprenaline and esmolol were unsuccessful, and the procedure was abandoned.

**Figure 2:**
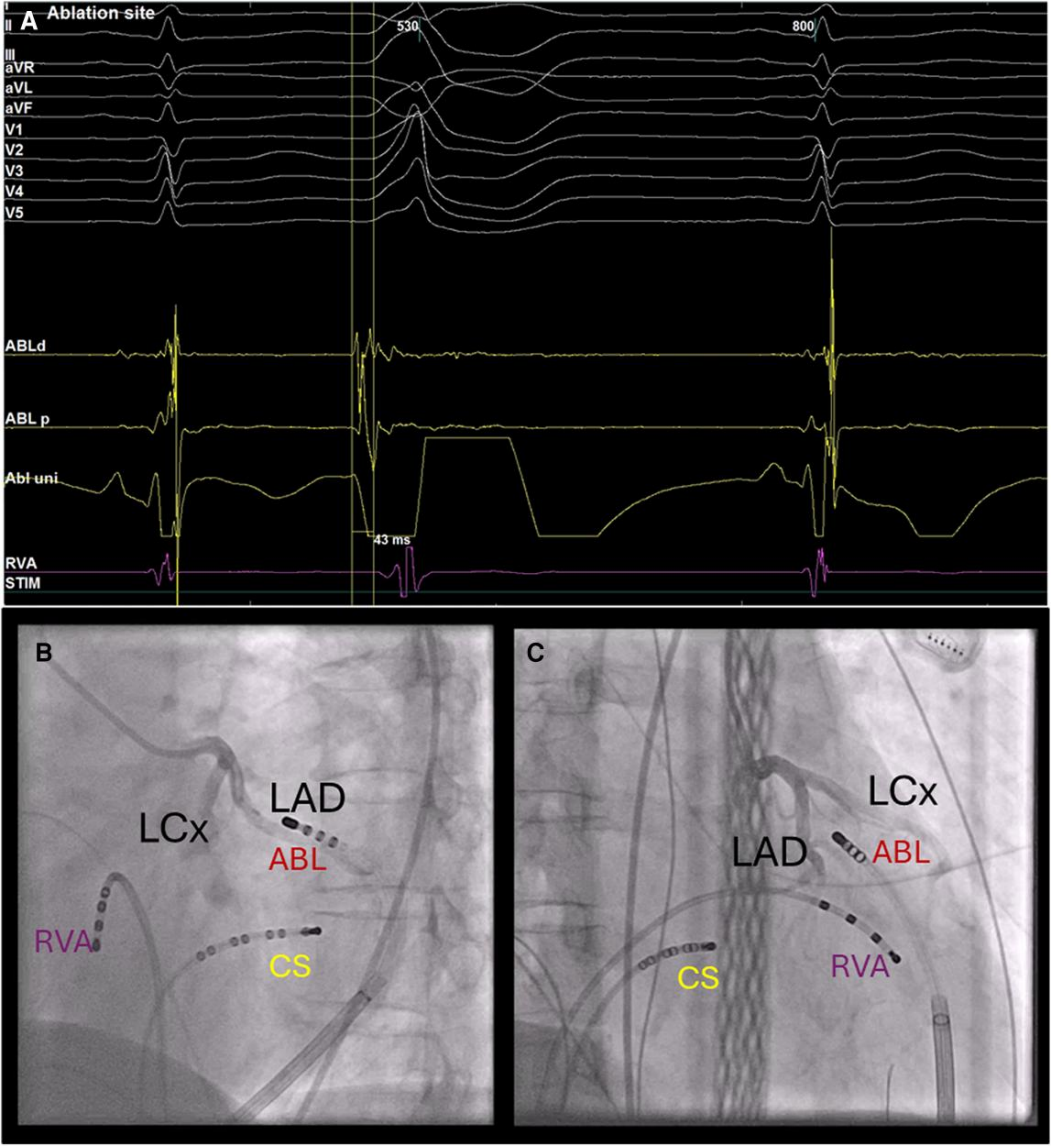
(*A*) Electrocardiogram, intracardiac, and unipolar signals from the catheters of the premature ventricular complex (second complex) at the site of successful ablation. The ablation catheter is epicardially positioned at the location of the premature ventricular complex; hence, the ablation catheter distal is the first lead where the premature ventricular complex is visible. (*B* and *C*) Coronary angiography (*B*, right anterior oblique view with 30° angle; *C*, left anterior oblique view with 60° angle) before the application of the pulsed field ablation. Ablation catheter is in proximity to the left coronary arteries. ABL, ablation catheter; ABLd, ablation catheter distal; ABLp, ablation catheter proximal; ABLuni, ablation catheter unipolar signal; CS, coronary sinus catheter; LAD, left anterior descending artery; LCx, left circumflex; RVA, right ventricular apex catheter.

A third procedure was started with local anaesthesia only to minimize the suppression of the PVC. A mapping catheter (EPstar Fix 2F, Japan Lifeline, Japan) was placed into the great cardiac vein to identify the location of the earliest signal (40 ms before QRS onset). However, an attempt to advance the ablation catheter (TactiCath™, Abbott Laboratories, IL, USA) through the great cardiac vein to the point of earliest activity was unsuccessful. A decision to use a subxiphoid epicardial approach was made. General anaesthesia was induced with ketamine and propofol. The PVC was initially suppressed, but successfully reinduced with 2 µg/min isoprenaline. Subxiphoid epicardial access was obtained under fluoroscopy, and a steerable sheath and ablation catheter (TactiCath™, Abbott Laboratories, IL, USA) were introduced. The PVC was mapped, and the earliest signal was found (45 ms before QRS onset, *Figure 2A*). Coronary angiography showed no abnormalities but indicated that the ablation catheter was in proximity to the CAs (*Figure 2B* and *C*, Supplementary material online, *Video S1*). We decided to use PFA, considering that RFA would most likely be ineffective due to the epicardial fat layer and unsafe due to the proximity to the CAs.

A 25A PFA pulse (CENTAURI™, Galaxy Medical, FL, USA) was applied with adequate pressure to the myocardial wall. Prior to the pulse, 400 µg nitro-glycerine was administered intravenously to prevent coronary spasm.^8^ A coronary angiography showed no abnormalities after PFA. Subsequent ventricular burst pacing with and without isoprenaline induced no further occurrences of PVCs.

**Figure 3:**
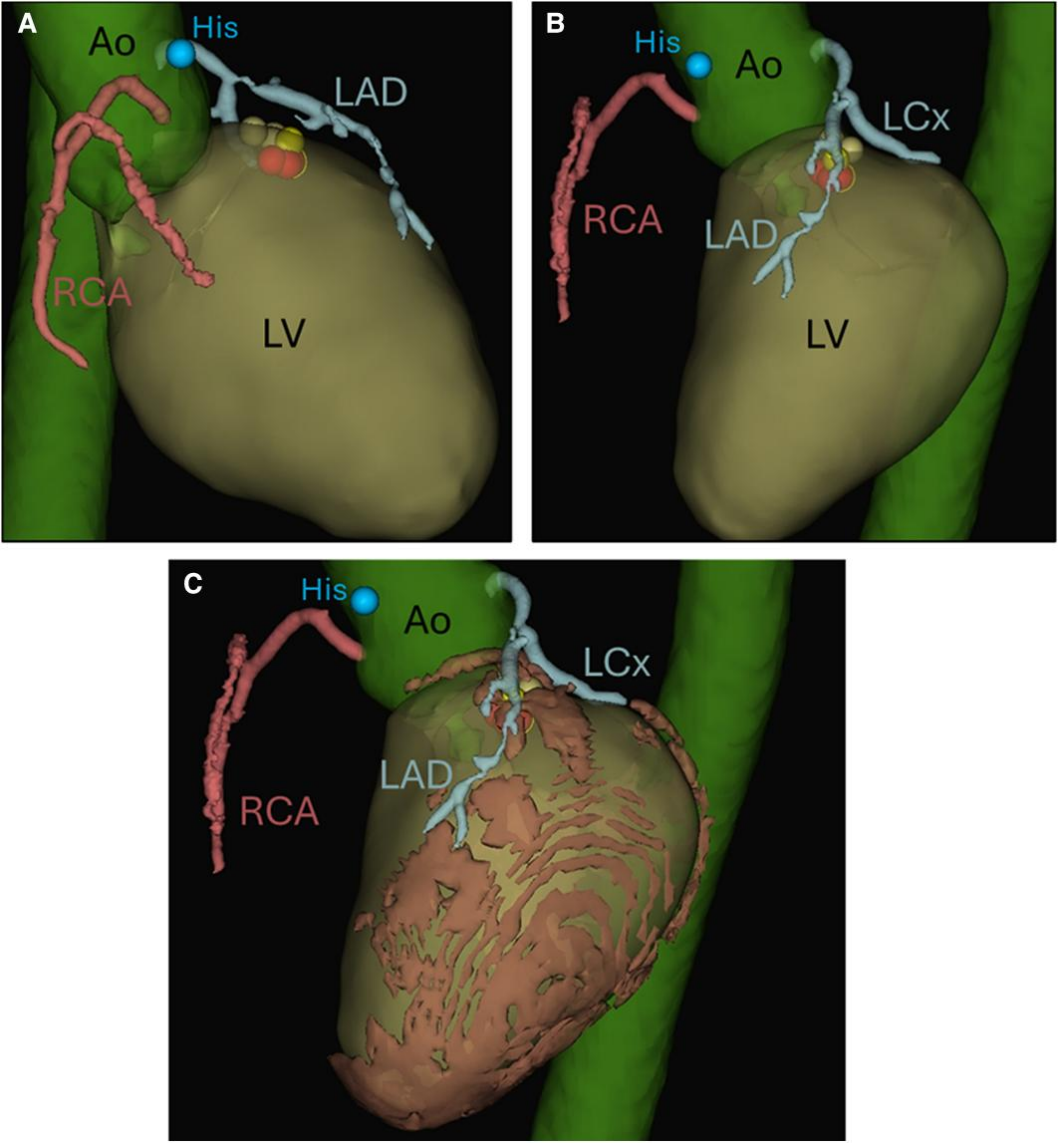
Post-procedure segmentation (*A*, right anterior oblique view with 30° angle; *B* and *C*, left anterior oblique view with 60° angle) of the coronary arteries, aorta, and left ventricle. The location of the His bundle is marked, the dots in the superior area of the LV indicate the ablation location of the premature ventricular complex. (*C*) Inclusion of the additional segmentation of the epicardial fat layer around the left ventricle. Ao, aorta; CA, coronary artery; His, location of the His bundle; LAD, left anterior descending; LCx, left circumflex; LV, left ventricle; PVC, premature ventricular complex; RCA, right coronary artery.

Post-procedure segmentation of the cardiac computed tomography (CT) with ADAS 3D segmentation software (ADAS 3D LV, ADAS 3D MEDICAL, Spain) aligned in the electrophysiology study (EnSite X EP System, Abbott) indicated that the PVC focus was indeed near the CAs and shielded by an epicardial fat layer (*Figure 3*). Computed tomography was obtained pre-procedurally with a pulmonary vein protocol, resulting in late phase imaging of the CAs, which manifests as insufficient contrast filling of the CAs. Angiography during procedure showed no stenosis (*Figure 2B* and *C*). The ADAS 3D system was not available at the time of the procedure and was therefore only used after the procedure.

A 6-month follow-up electrocardiogram (*Figure 1B*) showed a sinus rhythm. Since the final procedure, the patient was asymptomatic and reported significant improvements in quality of life.

## Discussion

We describe a unique case treating PVCs originating from an epicardial LV summit focus with PFA instead of RFA. To our knowledge, no such case has been reported yet.

The treatment with catheter ablation of LV summit focus PVCs is challenging due to the presence of epicardial fat and proximity to CA.^3^ Baman *et al*.^11^ found that 15% of idiopathic ventricular arrhythmias had an epicardial focus, of which 70% could be ablated from the coronary venous system. In the remaining 30% of cases, a subxiphoid approach is required. However, in about two-thirds of these cases, RFA is not possible due to nearby CAs.^5^

Pulsed field ablation was originally intended as an ablation modality for atrial fibrillation.^12^ Pulsed field ablation was later shown to be safe for epicardial application near the CA and reliable for the creation of long-lasting lesions through the epicardial fat layer.^9,10^ Higuchi *et al*.^13^ demonstrated in a porcine model that PFA applied directly on the CA was associated with mild-grade stenosis. Application of PFA near the CAs is also associated with coronary spasm; however, this can be avoided by pre-administration of nitro-glycerine.^8^ During our procedure, no coronary spasms were detected.

## Conclusion

A 45-year-old patient with symptomatic epicardial LV summit PVCs underwent successful PFA via a subxiphoid approach, where RFA could not be used due to the proximity of the CAs. We conclude that PFA might be a feasible option for catheter ablation of PVCs originating from an epicardial focus, in particular the LV summit.

## Supplementary Material

ytae478_Supplementary_Data

## Data Availability

The data underlying this article will be shared on reasonable request to the corresponding author.

## Lead author biography

Biomedical engineering graduate at the University of Twente and currently an imaging engineer at the HAGA Teaching Hospital.

## Supplementary material

**Consent:** The authors confirm that written consent for submission and publication of this case report including images and associated text has been obtained from the patient in line with COPE guidelines.

**Funding:** None declared.
